# The Muco-Microbiotic Layer in Respiratory Pathophysiology: Integrating Transcriptomics, Inflammation Phenotypes, and Clinical Biomarkers in Precision Pulmonology

**DOI:** 10.3390/biology15090684

**Published:** 2026-04-27

**Authors:** Claudio Candia, Adelaide Carista, Melania Ionelia Gratie, Domiziana Picone, Giuseppa D’Amico, Celeste Caruso Bavisotto, Fabio Bucchieri, Silvestro Ennio D’Anna, Alessandro Pitruzzella, Mauro Maniscalco, Giuseppe Bonaventura, Stefano Burgio, Francesco Cappello

**Affiliations:** 1 Istituti Clinici Scientifici Maugeri IRCCS, Pulmonary Rehabilitation Unit of Telese Terme Institute, 82037 Telese Terme, Italymauro.maniscalco@icsmaugeri.it (M.M.); 2 Department of Biomedicine, Neurosciences and Advanced Diagnostics (BiND), University of Palermo, 90127 Palermo, Italydomiziana.picone@unipa.it (D.P.); giuseppa.damico01@unipa.it (G.D.); celeste.carusobavisotto@unipa.it (C.C.B.); francesco.cappello@unipa.it (F.C.); 3 Department of Advanced Biomedical Sciences, University of Naples “Federico II”, 80131 Naples, Italy; 4 Department of Medicine and Surgery, Kore University of Enna, 94100 Enna, Italy; 5 Euro-Mediterranean Institute of Science and Technology, 90139 Palermo, Italy

**Keywords:** muco-microbiotic layer, lower airways, transcriptomics, metatranscriptomics, extracellular vesicles, airway microbiota, inflammatory phenotypes, precision pulmonology

## Abstract

The lower airways are a dynamic environment where physical, microbial, and molecular factors intersect to regulate respiratory health and disease. The muco-microbiotic (MuMi) layer, composed of mucus, resident microbes, and extracellular vesicles (EVs), is not just a passive barrier but also an active site for host–microbe communication. This layer integrates epithelial cell biology, microbial activity, and immune responses within the bronchial environment. New transcriptomic and metatranscriptomic technologies show that it is not only which microbes are present but also their gene activity that closely links to airway inflammation and disease. EV-associated RNAs from both host and microbial cells act as key messengers, influencing epithelial responses, immune activity, mucus properties, and microbial behaviour. This review highlights evidence that positions the MuMi layer as central to understanding lower airway disease, particularly asthma and chronic obstructive pulmonary disease (COPD). Distinct gene expression programs and biomarker profiles, such as exhaled nitric oxide, may reflect different disease mechanisms even in cases with similar clinical features, such as eosinophilia. Multi-omic approaches focused on the MuMi layer enable better disease classification, biomarker discovery, and therapy selection. By putting the MuMi interface at the core of precision pulmonology, we provide a framework for advancing personalised care in chronic respiratory diseases.

## Simple Summary

We propose that the lower airways, as well as the gastroenteric tract, host a complex environment known as the muco-microbiotic (MuMi) layer. This layer is a mixture of mucus, microbes, and tiny cellular particles. It functions as more than a protective shield. It also functions as an active communication center. Here, the body’s cells, immune system, and resident bacteria interact. Recent advances in technology have shown that lung health is determined not only by which microbes inhabit it but also by their specific gene activity, which drives inflammation and disease. This layer uses “extracellular vesicles” to ferry genetic and epigenetic messages between the host and microbes. These messages influence everything from immune response to mucus thickness. Understanding this interface may become central to the treatment of diseases such as asthma and COPD. It helps explain why patients with similar symptoms often require different treatments. By focusing on this specific layer, doctors aim to advance “precision pulmonology.” This approach moves away from one-size-fits-all therapies toward personalised care based on the unique biological activity in a patient’s airways.

## 1. Introduction

In recent years, it has been proposed that a distinct morphofunctional compartment exists within specific hollow organs that are directly or indirectly exposed to the external environment—the muco-microbiotic (MuMi) one—representing the interface where mucus, resident microbiota, and extracellular vesicles (EVs) interact to maintain local homeostasis as well as influence disease onset and progression [1,2,3]. Importantly, the MuMi layer should not be interpreted as a separate anatomical organ, but rather as the most internal biologically active functional layer of the hollow organ’s wall surface. The lack of earlier descriptions of the MuMi layer is largely attributable to the use of conventional tissue processing techniques, which, by using organic solvents—primarily alcohol and acetone—cause mucus solubilization and loss. This artefact leads to dispersion of the microbial component and prevents visualisation of the intact layer at the microscopic level.

This concept was first introduced and characterised in the gastrointestinal tract, where the so-called MuMi layer was described as the morpho-functional compartment located at the immediate luminal surface of these hollow organs, forming a dynamic and biologically active ecosystem in which epithelial and microbial nanovesicular components (such as EVs) and soluble factors engage, through the interposition of mucus, in reciprocal molecular communication [1,2,3]. Building on these foundations, we propose that the same structural and functional model can be applied to the respiratory system, encompassing both upper and lower airways.

From an embryological perspective, the lower airways originate as a pharyngeal diverticulum from the primitive foregut. This shared origin explains why the innermost respiratory and alimentary layers are morphologically and functionally similar. They later differentiate according to their specific physiological requirements [4,5]. Thus, the recognition of a MuMi interface in the respiratory tract also receives strong developmental support.

Anatomically, the wall of the lower airways can be described, from the lumen outward, as several layers in sequence. The MuMi layer consists of mucus, resident microbiota, and extracellular vesicles (EVs) derived from both epithelial cells (e.g., exosomes) and microbes (e.g., outer membrane vesicles, OMVs). This layer is not a separate anatomical structure and does not replace the mucosa. Instead, it is the innermost biologically active compartment of the wall and is directly exposed to the airway lumen.

Beneath it lies the respiratory mucosa, which includes a ciliated pseudostratified epithelium with ciliate and goblet cells as well as other epithelial cytotypes (e.g., basal or undifferentiated cells, essential for continuous epithelial renewal and tissue remodelling; Kulchitsky cells, enterochromaffin elements with neuroendocrine functions). The lamina propria, located beneath the epithelium, consists of loose connective tissue rich in vessels and immune cells, and the two are separated by a thin basement membrane. Deeper still, the intermediate (fibro-musculo-cartilaginous) layer, characterised by fibrous connective tissue, smooth muscle, and predominantly hyaline cartilage, provides mechanical support and participates in the regulation of airway calibre. The outermost adventitia anchors the bronchial wall to surrounding tissues [6].

It should be emphasised that numerous clinical studies on airway pathophysiology improperly use the term “submucosa” to refer to the connective tissue lying beneath the epithelium. Although this terminology has become widespread in the respiratory field, it is correct to point out that a true “submucosa”—in strict anatomical and histological terms—exists only in the alimentary canal, where it separates the mucosa from the muscularis propria. In scientific environments where precision in terminology is essential for clear, unambiguous communication, maintaining accurate anatomical terminology is particularly important.

The mucus that coats the respiratory mucosa performs essential protective and rheological functions, facilitating mucociliary clearance and forming the first biochemical barrier against inhaled agents. Alterations in its composition, concentration, or viscoelastic properties are implicated in muco-obstructive diseases, including asthma, chronic obstructive pulmonary disease (COPD), cystic fibrosis, and other respiratory diseases [7,8]. Within this mucus, however, lies a complex microenvironment—the respiratory microbiota—where microbial and epithelial cells interact dynamically via soluble molecules and, above all, EVs.

Recent research has demonstrated that the respiratory microbiota actively shapes immune tone and mucosal homeostasis, and that its functional transcriptional activity—rather than mere taxonomic composition—correlates with disease phenotypes [9]. Moreover, EVs produced by both host and microbial cells act as vehicles for proteins, lipids, and RNAs, mediating intercellular and even inter-kingdom communication [10]. These nanovesicles can cross the mucus barrier and deliver regulatory messages to epithelial or immune target cells, influencing homeostasis, inflammation, regeneration, and tolerance mechanisms.

Understanding the airway surface, therefore, requires moving beyond static anatomical models towards dynamic, ecological, and molecular perspectives. In this context, omics approaches, such as transcriptomic and metatranscriptomic analyses, provide powerful tools for elucidating the functional activity of both host and microbial components, enabling the identification of active genes and signalling pathways involved in respiratory physiology and pathology [9,11]. Beyond its morphological definition, the respiratory MuMi layer represents a critical functional diagnostic target. Our previous work established its presence in the gut and the lungs. This interface acts as a real-time ‘molecular dashboard.’ By integrating EV-associated transcriptomics with clinical parameters, we can identify specific ‘muco-microbiotomic’ endotypes. This moves the field toward practical applications. It allows for tailoring biological therapies to a patient’s microbial–host crosstalk rather than relying on broad clinical phenotypes [12].

Clinically, integrating these molecular data with functional and inflammatory biomarkers—such as fractional exhaled nitric oxide (FeNO) and blood eosinophil count—may help explain the divergent inflammatory phenotypes observed in diseases such as asthma and COPD, as also suggested by recent findings [13].

**Figure 1:**
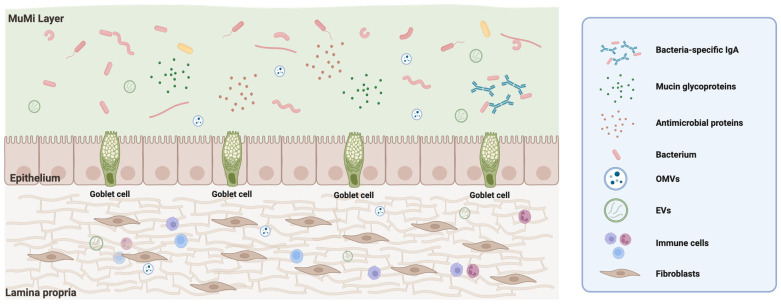
Representation of the MuMi layer. The image depicts the structural organisation of the muco-microbiotic (MuMi) layer in the airways, presenting it as a dynamic functional interface between the respiratory lumen and the underlying tissue. In the upper portion, the MuMi layer itself appears as a complex ecosystem in which mucus incorporates the resident microbiota, antimicrobial proteins, mucin glycoproteins and bacteria-specific IgA antibodies. Within this fluid matrix, a bidirectional exchange of extracellular vesicles (EVs) and outer membrane vesicles (OMVs) of bacterial origin can be observed, which act as vectors for molecular communication between host and microbes. Beneath this lies the respiratory epithelium, characterised by the presence of goblet cells specialised in secreting the mucus components necessary for maintaining this biochemical barrier. The structure rests on the lamina propria, a compartment of connective tissue that houses fibroblasts and immune cells, which actively interact with molecular signals and vesicles originating from the luminal surface. The image highlights that the MuMi is not an isolated anatomical structure but a functional compartment essential for local homeostasis and host defence. It should be noted that the biological components shown in the figure, such as EVs and OMVs, are not drawn to scale. Created with https://app.biorender.com/ (accessed on 2 March 2026).

This review aims to provide a comprehensive synthesis of current knowledge on the respiratory MuMi layer, addressing (i) its anatomical and molecular definition, (ii) the methodological advances that enable its characterization through transcriptomics and multi-omics approaches, (iii) the disease-specific transcriptional signatures linking MuMi-layer alterations to clinical phenotypes, and (iv) the translational implications of this emerging paradigm for precision respiratory medicine. In this perspective, the MuMi layer should be understood not as a discrete anatomical component, but as a dynamic ecological interface at the internal wall surface where host and microbial components interact and communicate (Figure 1).

## 2. The Airway Muco-Microbiotic Layer: Composition and Dynamics

In the respiratory tract, a resident microbiota persists even in healthy airways, although its biomass is markedly lower than in the gut. The bacterial community in this niche comprises phyla such as *Firmicutes*, *Proteobacteria*, *Bacteroidetes*, and *Actinobacteria*, and *genera* frequently identified include *Streptococcus*, *Prevotella*, and *Veillonella* [14].

Epithelial cells lining the airways—including ciliated and goblet cells—do not simply act as a passive barrier. They actively communicate with resident microbes via pattern-recognition receptors, thereby influencing innate and adaptive immune responses and contributing to local immune homeostasis. Dysregulation of this crosstalk, for instance, due to infection or inflammation, may drive pathological processes [14].

In this framework, we extend the concept of the MuMi layer to the airways: we propose that the mucous surface environment of the respiratory tract, particularly the mucus layer coating the epithelium, embeds not only commensal and, occasionally, pathogenic microbes (bacteria, viruses, fungi) but also EVs released by both host cells and the microbiota, forming a dynamic and biologically active ecosystem. This ecosystem enables bidirectional molecular communication mediated by EVs (including exosomes, microvesicles, and microbe-derived EVs, such as Gram-negative bacterial OMVs) [15,16].

Microbe-derived EVs appear to play a particularly important role in respiratory diseases. For example, in chronic respiratory conditions such as asthma and COPD, the profiles, abundances, and immunological impacts of bacterial EVs differ significantly from those in healthy individuals [17]. Moreover, EV-associated microRNAs (EV-miRNAs) contribute to intercellular signalling within the lung, modulating inflammation, epithelial repair, and immune responses [18]. The MuMi layer also functions as a complex physicochemical and metabolic interface. The airway environment is characterised by distinct local gradients of oxygen tension, pH, and temperature, which continuously shape microbial niches and their metabolic outputs. This metabolic exchange between host and resident community adds a functional dimension to the MuMi framework, positioning it as a dynamic hub of molecular exchange. Finally, dysbiosis attributable to components of the airway MuMi layer—alterations in microbial composition or in EV production—has been associated with the pathogenesis of respiratory diseases, including asthma and COPD [17,19].

Building on this structural, compositional and metabolic framework, investigating the functional activity of the airway MuMi layer through transcriptomic and metatranscriptomic approaches allows us to move beyond taxonomy, revealing microbial metabolism, host–microbe interactions, inflammatory signatures, and immunomodulatory pathways, insights that are increasingly informing our understanding of chronic respiratory diseases.

Finally, while the bacterial component of the airway microbiota has been most extensively studied, growing evidence indicates that the respiratory virome and mycobiome also contribute significantly to mucosal homeostasis and disease dynamics. Respiratory viruses—including rhinoviruses, influenza viruses, and coronaviruses—are well-recognised triggers of exacerbations in asthma and COPD, while fungal communities (e.g., *Aspergillus*, *Candida*, and *Malassezia*) have been implicated in airway inflammation, allergic sensitisation, and chronic lung disease [9,11,17]. Within the MuMi framework, these components can be viewed as additional participants in the mucosal ecological network, interacting with host cells and bacterial communities through molecular signalling, metabolic exchanges, and immune modulation.

## 3. Sampling MuMi Layer: A Brief Overview

As the MuMi layer corresponds to the functional luminal compartment of the airway mucosal surface, it is potentially accessible through both invasive and non-invasive sampling techniques. Among invasive approaches, bronchoalveolar lavage (BAL) fluid sampling is the most common, having been used since the 1970s to characterise several respiratory conditions, including interstitial lung diseases [20]. Briefly, a known volume of sterile saline is instilled in two or three aliquots—typically into the right middle lobe—followed by gentle aspiration to recover the largest possible amount of fluid. The recovered volume is recorded to account for dilution. This technique is widely used in clinical practice to investigate the immunological and microbiological composition of the distal airways and has also been employed in several metabolomics studies to characterise disease phenotypes, especially within the clinical setting of COPD [21,22]. While BAL is likely the most direct method for accessing the MuMi layer within the airways, it is technically demanding, requiring trained operators, specialised equipment, and experienced laboratory personnel for sample processing. Moreover, because of its invasive nature, BAL can only be performed in selected patient populations. However, when considered specifically in the context of MuMi interface analysis, BAL presents important limitations. The instillation of relatively large volumes of saline dilutes the native mucus layer and disrupts its spatial organization, potentially washing away loosely adherent microbes, EVs, and soluble mediators that normally participate in the mucosal ecological network. As a consequence, BAL samples may provide a representative overview of airway cellular and molecular components but may not fully preserve the structural and functional integrity of the MuMi interface in situ [23].

The most widely used noninvasive method for sampling airway lining fluid is exhaled breath condensate (EBC) [24]. EBC is a biological matrix composed predominantly of water (approximately 99.9%), but it also contains a variety of aerosolised molecules, including volatile organic compounds, inorganic nonvolatile molecules, pollutants, and small proteins, which can be analysed using various omics approaches, such as mass spectrometry or nuclear magnetic resonance-based metabolomics. In brief, the patient breathes through a closed circuit that passes through a cooling chamber, allowing exhaled air to condense. Several research teams have employed EBC to diagnose respiratory conditions [25,26], identify respiratory disease phenotypes [27], study environmental exposures to pollutants [28], and monitor the airway response to biologic treatment [25,29,30]. Although this technique is safe and relatively inexpensive, EBC presents several challenges related to standardisation, storage, and analytical procedures, limiting its applicability in routine clinical practice. However, in the context of MuMi interface investigation, EBC may have important limitations: because the collected condensate consists predominantly of water with very low cellular or particulate content, it does not adequately capture the microbial communities, EVs, or structural components of the mucus-associated microenvironment. Consequently, EBC is better suited for analysing volatile compounds and soluble inflammatory mediators rather than for direct characterisation of the MuMi ecosystem.

Finally, induced sputum has gained renewed interest in recent years as a validated and clinically useful method for sampling deep airway secretions [31]. In this procedure, the patient inhales a hypertonic saline solution delivered by ultrasonic nebulization, which induces coughing and facilitates the expectoration of airway secretions [32]. The collected sample is then processed to separate the cellular component from the supernatant, thereby enabling the study of lung cytology and the quantification and identification of cytokines, pollutants, and EVs [33,34]. The main limitation of this approach is its time-consuming nature, as it typically requires pretreatment with bronchodilators and repeated spirometric assessments to monitor lung function throughout the procedure. Moreover, compared with BAL, this approach samples mucus originating from the bronchial surface with considerably less dilution, thereby potentially preserving a greater proportion of the native mucus-associated microbial and vesicular components. For this reason, induced sputum may be particularly informative for investigating the MuMi, as it captures material directly from the airway mucosal surface, where host cells, microbes, and EVs interact [35]. Although contamination from the upper airways remains a recognised limitation, appropriate processing and analytical approaches can mitigate this issue and allow meaningful insights into MuMi-related processes.

## 4. Transcriptomics and Metatranscriptomics: Unveiling Functional Activity

In recent years, the study of the microbiotic composition in the respiratory tract has evolved beyond merely cataloguing which microbes are present: metagenomics permits us to identify the genetic potential of microbial communities (i.e., what they could do), but metatranscriptomics reveals which genes are actually being expressed at a given time, in response to the local environment [36]. This distinction is significant within the airways, which function as regional filters and establish a physiological landscape characterised by gradients in oxygen levels, pH, and temperature that decrease from the upper to lower regions of the lungs. These variables, together with the quality of inhaled air and the availability of nutrients such as mucins and lipids, can fluctuate rapidly and affect both the metabolic activity of resident microbes and the epithelial response [37].

In particular, metatranscriptomic analyses of respiratory samples have revealed remarkable functional plasticity within the microbial community. Active microbial metabolism often involves pathways linked to fermentation (for example, lactate production), redox regulation, and the synthesis of small bioactive metabolites, such as short-chain fatty acids and amino derivatives, which can, in turn, modulate mucosal viscosity and airway epithelial physiology [38]. This metabolic activity is closely associated with the immunological set point of the lung, in which low-biomass microbial signals activate innate immune pathways without inducing overt inflammation. Simultaneously, transcriptional signatures associated with inflammation (e.g., NF-κB, interferon-stimulated genes) and cellular stress have been observed, alongside changes in host transcript expression of mucin genes (e.g., *MUC5AC, MUC5B*) and other defence or remodelling pathways [39]. Moreover, immunoregulatory transcripts—such as those involved in the IL-10 axis and in intercellular signalling—suggest that the MuMi layer is not a passive bystander but an active contributor to shaping the mucosal immune tone.

Emerging metatranscriptomic studies in chronic respiratory diseases reinforce this functional paradigm. For example, in COPD, metatranscriptomic analysis of BAL fluid has shown that transcriptionally active *Streptococcus*, *Rothia*, or *Pseudomonas* species correlate with high bacterial biomass, stronger Th17 immune responses, and more frequent exacerbations [40]. In bronchial biopsies from stable COPD patients, ultra-low levels of microbial RNA were detectable, and their presence was linked to host unfolded protein response pathways and microRNA-155 expression [40]. A comprehensive multi-omic meta-analysis of COPD sputum and transcriptomic data further identified microbial metabolites (e.g., butyrate, homocysteine, and palmitate) that interact with host gene signatures, suggesting synergistic microbe–host metabolic crosstalk [41]. More recently, a longitudinal study of smokers with mild-to-moderate COPD using host and microbial RNA sequencing showed that lower airway dysbiosis, enriched in oral commensals, is associated with inflammatory transcriptomic signatures (e.g., IL-17, IL-6, PI3K, mucin genes), suggesting that dysbiosis contributes mechanistically to early airway injury [42].

In asthma, integrative studies combining nasal and bronchial microbiomes with host transcriptomes have revealed associations between specific genera (e.g., *Moraxella*, *Alloiococcus*) and host genes involved in inflammatory or ciliary function, suggesting protective or pathogenic roles [43]. In addition, metatranscriptomic methods applied to low-biomass clinical samples have successfully captured the respiratory virome, active bacterial and fungal communities, and the host immune response, highlighting, for instance, upregulation of innate immune and inflammasome pathways in viral infection [44].

These findings collectively reinforce the view that the MuMi layer is not merely a “witness” of inflammation but a functionally active actor that dynamically modulates its environment. The transition from a healthy “steady-state” to disease is often marked by a breakdown in these homeostatic interactions, in which environmental irritants or pathogens shift microbial metabolic output toward pro-inflammatory signatures. The clinical relevance of metatranscriptomic insights is becoming evident: by combining expression-based microbial data with host transcriptomics, proteomics, and clinical biomarkers, it may be possible to define functional endotypes of disease. Such stratification could then guide precision interventions, e.g., the rational use of immunomodulatory antibiotics, targeted modulation of the microbiota, or therapies aimed at restoring mucin balance based on an individual’s transcriptomic profile.

Importantly, metatranscriptomic approaches also capture the functional activity of the airway virome and mycobiome. This is particularly relevant in respiratory diseases, where viral infections frequently precipitate acute exacerbations and where fungal colonisation may contribute to chronic inflammatory responses. Within the MuMi framework, these components are not merely transient occupants of the airway lumen but integral elements of the mucosal ecological network, whose transcriptional activity can influence epithelial signalling pathways, immune tone, and microbial community dynamics [11,17].

Table 1 summarises emerging metatranscriptomic signatures in chronic respiratory diseases. Taken together, these metatranscriptomic signatures underscore the need to look beyond intracellular RNA expression to extracellular routes of communication, particularly the RNA species packaged within EVs.

## 5. Extracellular Vesicle-Associated Transcriptomics: RNA-Mediated Crosstalk

**Figure 2:**
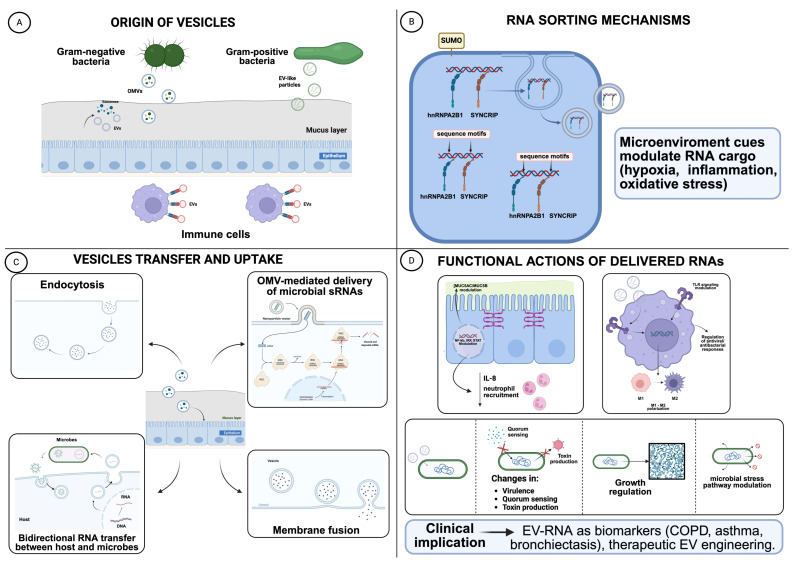
Mechanisms of RNA Sorting, Vesicle-Mediated Transfer, and Functional Actions in the Respiratory Muco-Microbiotic Interface. Schematic representation of extracellular vesicle (EV)-associated RNA communication within the respiratory mucosal microenvironment. (**A**) Diverse cell types contribute to the EV pool in the airways, including epithelial and immune cells, which release exosomes and microvesicles, as well as microorganisms that shed outer membrane vesicles (OMVs) or EV-like particles. These vesicles traverse the mucus layer and encapsulate complex RNA cargos reflecting the physiological or pathological state of the producing cell. (**B**) RNA loading into EVs is governed by selective sorting mechanisms rather than passive encapsulation. RNA-binding proteins (RBPs), such as hnRNPA2B1 and SYNCRIP, recognise specific sequence motifs within miRNAs and other small RNAs and direct them toward vesicular compartments. Post-translational modifications, including SUMOylation, together with microenvironmental cues (e.g., hypoxia, inflammation, oxidative stress), dynamically modulate RNA selection and EV composition. (**C**) EVs migrate across the mucus barrier and deliver their RNA cargo to recipient cells through multiple uptake routes, including endocytosis and membrane fusion. Microbial OMVs efficiently transfer bacterial small RNAs to airway epithelial cells, while host-derived EVs can also target microbes, enabling bidirectional, inter-kingdom RNA exchange within the respiratory niche. (**D**) Once delivered, EV-associated RNAs exert pleiotropic functional effects. In epithelial cells, they modulate inflammatory signalling, barrier integrity, and mucin production; in immune cells, they shape innate and adaptive responses; and in microbial communities, they influence virulence, quorum sensing, metabolic activity, stress responses, and growth. Collectively, these EV-mediated RNA circuits contribute to host–microbe homeostasis and disease-associated remodelling of the respiratory mucosal interface, with important diagnostic and therapeutic implications. Created with https://app.biorender.com/ (accessed on 8 January 2026).

EVs constitute an additional, dynamic, and tightly regulated level of transcriptomic communication within the respiratory microenvironment, where signalling must occur rapidly, selectively, and across mucus barriers. EVs are a heterogeneous population that includes apoptotic bodies, microvesicles, and exosomes, released by both eukaryotic cells (epithelial, immune, endothelial, and stromal cells) and microorganisms (OMVs from Gram-negative bacteria; other vesicles from Gram-positive bacteria and fungi) [10] (Figure 2A). Indeed, EVs within the lower airway mucosal environment may originate from both host cells and microorganisms. Lower airway epithelial cells and immune cells actively release vesicles involved in intercellular signalling, while bacteria can produce membrane vesicles capable of transporting proteins, lipids, and nucleic acids. These vesicle populations may interact within the MuMi layer, potentially mediating cross-kingdom communication between host and microbiota [46,47].

In chronic inflammatory contexts, these host-derived EVs have emerged as highly stable, non-invasive “liquid biopsies” present in BAL fluid and induced sputum, offering a window into the activation state of the airway epithelium and the underlying immune landscape [48]. These particles encapsulate complex cargos of lipids, proteins, and nucleic acids, including full-length or fragmented mRNAs, small RNAs, microRNAs, and long non-coding RNAs [49]. Their composition partly reflects the physiological or pathological state of the producing cell and enables the targeted delivery of regulatory molecules across the heterogeneous niches of the respiratory tract [50,51].

In experimental settings, RNA molecules associated with extracellular vesicles are typically distinguished from free extracellular RNA through vesicle isolation and purification procedures, such as differential ultracentrifugation or density gradient separation, often combined with EVs marker profiling. RNase protection assays are also commonly used to verify that the detected RNA is protected within vesicular structures [52,53].

The presence of RNA within microbial EVs—particularly OMVs—is now well established. Profiling studies have shown that bacterial OMVs selectively enrich regulatory small RNAs and tRNA fragments that can be transferred to airway epithelial cells and modulate host responses. A prominent example is sRNA52320, a methionine tRNA–derived fragment carried by *Pseudomonas aeruginosa* OMVs, which suppresses IL-8 secretion in bronchial epithelial cells and dampens neutrophil chemotaxis in vivo. These findings demonstrate not only the functional relevance of microbe-derived small RNAs but also the efficiency of OMVs as delivery vehicles for RNA-encoded immune modulation [54].

This vesicle-mediated RNA trafficking establishes a bidirectional, inter-kingdom dialogue that expands the canonical view of host–microbe interactions. Microbial EV-associated RNAs can influence epithelial barrier integrity, mucin production, inflammatory cascades, and stress responses [51]. Conversely, host-derived EVs—particularly exosomes and microvesicles from airway epithelial or immune cells—carry miRNAs and other RNA species that can modulate microbial gene expression, thereby affecting functions such as virulence, metabolic activity, and proliferation [55]. The well-documented concept of host-derived miRNAs entering microbes and reshaping bacterial transcription in the gastrointestinal tract provides a strong biological precedent for analogous mechanisms in respiratory mucosal ecosystems.

At the molecular level, RNA packaging into EVs is not stochastic but driven by selective sorting mechanisms (Figure 2B). RNA-binding proteins, such as hnRNPA2B1 and SYNCRIP, recognise specific sequence motifs within miRNAs and direct them toward exosomal loading [56]. These interactions are modulated by post-translational modifications—including SUMOylation—which fine-tune the selective enrichment of particular RNA species. Such sorting rules are critical for understanding why certain miRNAs or small RNAs are preferentially transported and therefore disproportionately represented in intercellular or inter-kingdom communication [57].

The microenvironment strongly shapes both the quantity and the molecular composition of released EVs. Conditions such as hypoxia, inflammation, oxidative stress, nutrient fluctuations, and altered mucus rheology can remodel EVs’ output and influence RNA cargo selection. For example, hypoxic or injury-associated states shift EV profiles toward miRNAs involved in inflammation, repair, and metabolic adaptation [50]. Given the marked gradients in oxygen tension, pH, microbial abundance, and immune activity along the respiratory tract, EV-mediated RNA exchange likely functions as a dynamic buffer system that aligns epithelial, immune, and microbial behaviour with rapidly changing environmental cues.

The functional implications of these processes are profound. EV-associated RNA profiles can serve as dynamic biomarkers of inflammatory phenotypes (e.g., COPD, asthma, chronic infections), epithelial remodelling, or microbe–host interactions (Figure 2D). EV-mediated transfer of miRNAs to epithelial targets may influence mucus production, barrier regulation, and innate responses, whereas microbial OMVs protect their RNA content from extracellular RNases and facilitate targeted uptake by host cells, thereby enabling long-range regulatory influence across the mucosal surface [51] (Figure 2C). Moreover, EV-mediated RNA signalling may contribute to microbial competition within the respiratory niche by modulating stress responses, quorum-sensing pathways, or antimicrobial resistance mechanisms [54].

From a methodological standpoint, EV-associated transcriptomic analysis—whether using BAL fluid, induced sputum, nasal lavage, or in vitro airway models—requires optimised enrichment protocols, rigorous contamination control, and adherence to international guidelines to ensure biological specificity and reproducibility [10]. Integrating EV-RNA profiles with metatranscriptomic signals and inflammatory phenotypes could support the development of multidimensional biomarkers that stratify clinical endotypes and guide precision therapies [50]. Furthermore, leveraging engineered EVs as delivery systems for therapeutic RNA represents an emerging translational opportunity in respiratory medicine [16].

Collectively, EVs–associated transcriptomics reveals a dynamic regulatory system that orchestrates microbe–host communication within the mucosal interface. Understanding how these vesicle-mediated RNA networks integrate with broader transcriptional programs in the airways is crucial, as disease-specific transcriptomic signatures increasingly define distinct inflammatory phenotypes and clinical trajectories. As illustrated in Figure 2, these EV-derived RNA circuits interface with key host pathways; the next section explores how these signatures manifest across major airway disorders and how they may inform precision diagnostic and therapeutic strategies.

## 6. Disease-Specific Transcriptomic Signatures in Airway Disorders

Transcriptomic profiling has revealed that airway diseases sharing similar clinical or inflammatory phenotypes may nonetheless be driven by distinct molecular programs. These disease-specific signatures arise from the integration of epithelial, immune, and microenvironmental cues and can uncover biological heterogeneity that is not apparent from conventional clinical classification. Notably, overlapping phenotypes, such as eosinophilic inflammation, may reflect divergent transcriptional architectures, with important implications for endotype definition, biomarker interpretation, and therapeutic response. In the following sections, we discuss how transcriptomic analyses have refined molecular stratification across major airway disorders and related pulmonary conditions.

### 6.1. Asthma and COPD: Divergent Transcriptomic Landscapes of Eosinophilic Inflammation

Despite similar clinical eosinophilic phenotypes in eosinophilic COPD (eCOPD) and eosinophilic severe asthma (eSA), underlying molecular programs may diverge significantly. A retrospective observational study by Candia and colleagues reported that, in patients stratified by peripheral eosinophil count ≥ 300 cells/µL, FeNO levels differed significantly between eCOPD and eSA, suggesting divergent underlying airway inflammatory biology even when eosinophilia appeared clinically similar [13].

Though Candia et al. did not perform RNA-sequencing themselves, transcriptomic studies of eosinophils and airway cells in COPD vs. asthma identify gene expression differences consistent with divergent T2 pathways, i.e., (a) an exploratory RNA-seq study comparing peripheral eosinophils from mild-to-moderate asthma vs. COPD patients identified 26 differentially expressed genes, with reduced expression of IL-4/IL-13 signaling genes in COPD eosinophils relative to asthma, implying distinct inflammatory regulation despite shared eosinophilia [58], and (b) another independent research in asthma identified epithelial genes correlating with eosinophilia and FeNO, including CDH26 among others, regulated post-allergen exposure, linking epithelial transcriptomic changes to T2 pathways in asthma [59]. This divergence is likely amplified by functional processes occurring at the MuMi interface, in which the microbiota’s metabolic output differentially primes the epithelial ‘set point’ in asthma compared with COPD. Collectively, these findings suggest that eosinophilic inflammation in COPD and severe asthma may involve distinct transcriptomic signatures, with T2-associated genes and pathways (e.g., IL-4/IL-13 signalling) differentially regulated in the two diseases, even when clinical eosinophil counts overlap. In COPD, the T2 profile is often less pronounced and may be coupled with other inflammatory pathways that influence FeNO production, miRNA profiles, and corticosteroid responsiveness, underscoring the need to consider the transcriptomic context when defining phenotypes. The differential expression of genes involved in immune signalling, tissue remodelling, and cytokine responses supports a model in which the clinical eosinophilic phenotype masks deeper transcriptional heterogeneity between eCOPD and eSA.

### 6.2. Other Respiratory Disorders

#### 6.2.1. Idiopathic Pulmonary Fibrosis (IPF)

IPF is characterised by progressive fibrosis and distinct molecular signatures reflective of extracellular matrix remodelling, immune dysregulation, and profibrotic signalling. Although comprehensive transcriptomic profiling of MuMi interface remains an emerging area, multi-omics and integrative analyses have highlighted networks involving TGF-β family members, fibroblast activation, and complement pathways that correlate with fibrotic progression and clinical severity [60].

#### 6.2.2. COVID-19 and Other Post-Infectious Conditions

Acute SARS-CoV-2 infection can profoundly affect the MuMi layer and systemic transcriptomes, particularly within immune cell populations such as neutrophils, where pathways related to immune regulation, autophagy, and chromatin modification are differentially expressed in severe disease [61]. Comparative transcriptomic analyses suggest overlapping yet disease-specific signatures between COVID-19 and other chronic lung conditions, including dysregulated inflammatory gene modules and EV-associated signalling networks that may persist or evolve during post-acute sequelae [62,63].

Influenza A virus (IAV) infection profoundly interacts with the respiratory mucosal environment. IAV induces robust host immune responses detectable at the proteomic and transcriptomic levels across species, reflecting antiviral signalling (e.g., interferons, cytokines) and inflammation, which, in turn, can alter mucosal integrity and mucus production [64]. Systemic studies in human blood reveal that influenza severity correlates with host genetic background and transcriptional programs governing interferon responses, myeloid activation, and immune regulation, suggesting that local airway events are tightly linked to broader immune states [65]. In parallel, post-transcriptional regulators such as microRNA-155 have been identified as key modulators of host defence, orchestrating IL-23/IL-17-dependent immune pathways that influence susceptibility to secondary bacterial pneumonia following viral infection [66]. Both in vivo and clinical omics studies show that viral infection disrupts epithelial homeostasis and can modify the respiratory microbiome, with reduced microbial diversity and increased abundance of certain bacterial taxa (e.g., *Firmicutes*, *Streptococcus*) associated with more severe disease, potentially affecting colonisation resistance and mucociliary clearance [67]. Dysbiosis of the airway microbiome may enhance susceptibility to secondary infections and exacerbate inflammatory injury, while commensal microbes modulate mucosal immunity and maintain barrier function [64]. These interactions underscore the importance of the muco-microbiotic layer as an active modulator of influenza pathophysiology, linking viral replication with host defence responses and microbial ecology at the airway surface.

Recent multi-omics and spatial transcriptomic analyses [68] in pediatric acute respiratory distress syndrome further support the muco-microbiotic layer as a key functional interface in lung injury and repair [69]. By integrating single-cell, spatial, and proteomic data from lung tissue, BAL fluid, and plasma, Song and collaborators [69] identified spatially confined epithelial repair niches characterised by preserved alveolar type II cells, differentiation toward alveolar type I cells, and activation of KRT17-associated stress-repair programs in underage patients with favourable outcomes. Importantly, these reparative signatures were accompanied by localised immune remodelling, including a shift from inflammatory to resident-like macrophage states, underscoring the coordinated interaction between epithelial, immune, and microenvironmental components at the airway surface. In contrast, fatal pediatric cases and adult severe lung injury exhibited diffuse inflammation, pro-fibrotic fibroblast activation, and impaired epithelial regeneration, suggesting a breakdown of localised MuMi-mediated homeostatic control. The detection of epithelial repair markers in airway fluids and the circulation further highlights how molecular events at the mucosal–microbiome interface can translate into measurable systemic signals.

#### 6.2.3. Occupational and Environmental Lung Diseases

**Figure 3:**
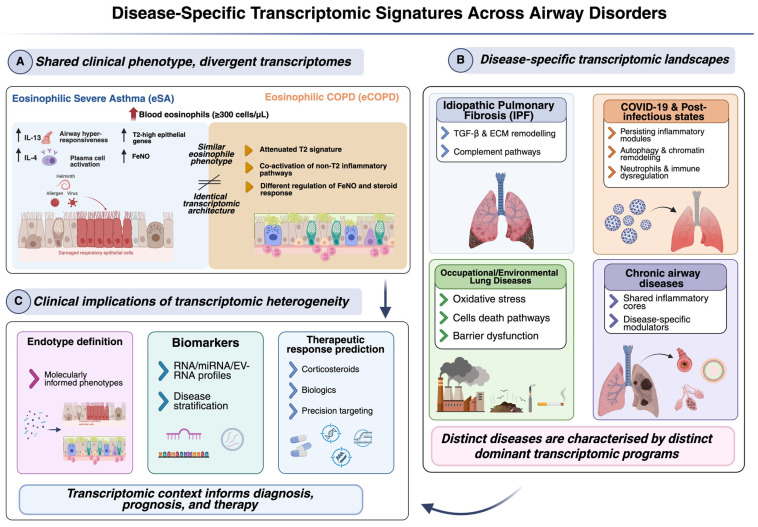
Disease-specific transcriptomic signatures across airway disorders. Schematic overview of how distinct airway diseases are characterised by disease-specific transcriptomic landscapes, even in the presence of overlapping clinical phenotypes. (**A**) Comparison of eosinophilic severe asthma (eSA) and eosinophilic COPD (eCOPD) illustrates that similar levels of blood eosinophilia can arise from divergent molecular programs. In eSA, eosinophilic inflammation is typically associated with a robust T2-high transcriptomic signature, including enhanced IL-4/IL-13 signaling, epithelial gene activation, and increased FeNO [59], whereas in eCOPD, the T2 profile is often attenuated and coupled with non-T2 inflammatory pathways, contributing to differential biomarker expression and corticosteroid responsiveness [72]. (**B**) Beyond asthma and COPD, distinct respiratory disorders display characteristic transcriptomic signatures, including profibrotic and extracellular matrix-remodelling pathways in idiopathic pulmonary fibrosis, immune dysregulation and chromatin remodelling in COVID-19 and post-infectious states, and oxidative stress- and cell death-related programs in occupational and environmental lung diseases. (**C**) These disease-specific transcriptomic profiles have direct clinical implications, informing molecular endotype definition, RNA- and EV-associated biomarker development, and the prediction of therapeutic responses. Collectively, the figure highlights how transcriptomic context refines disease classification and supports precision medicine approaches in airway disorders. Created with https://app.biorender.com/ (accessed on 18 January 2026).

Chronic exposure to inhaled toxicants (e.g., silica, asbestos, occupational dusts) can induce distinct transcriptomic changes in the MuMi layer, characterised by upregulation of stress-response genes, pro-inflammatory cytokines, and pathways mediating oxidative stress and cell death [70]. Additionally, environmental pollutants, such as particulate matter, have been associated with differential gene expression linked to immune activation and mucosal barrier function. These signatures reflect disease-specific remodelling of the mucosal transcriptome in response to chronic inhalational stress (Figure 3B) [71].

Together, these studies illustrate how clinically overlapping airway disorders are underpinned by distinct transcriptomic architectures that reflect disease-specific inflammatory programs, microenvironmental pressures, and remodelling processes (Figure 3A). As summarised in the Figure 3, transcriptomic profiling reveals both shared and divergent molecular signatures across asthma, COPD, fibrotic, infectious, and environmentally driven lung diseases, highlighting the importance of molecular context in MuMi layer for endotype definition, biomarker discovery, and precision therapeutic strategies (Figure 3A–C).

## 7. Integrative Multi-Omics and Translational Perspectives

Modern respiratory research is increasingly leveraging integrative multi-omics—the combined analysis of multiple omic layers, including metatrascriptomics, proteomics, lipidomics, metabolomics, and microbiomics—to dissect the molecular underpinnings of airway diseases and connect these to clinical phenotypes. These approaches enable systems-level views that transcend traditional single-omic or phenotype-only characterisation, proposing a path toward precision medicine in complex pulmonary disorders such as asthma and COPD [11,73].

A critical methodological issue in airway microbiome and transcriptomic studies is the potential contribution of microorganisms originating from the upper respiratory tract. To address this limitation, several strategies have been proposed, including optimised sampling procedures (e.g., protected specimen brushes or BAL), the use of appropriate negative controls during sequencing workflows, and bioinformatic pipelines specifically designed to identify and filter potential contaminant signals in low-biomass datasets [74,75].

Another critical aspect is the intrinsically low microbial biomass of lung samples. This condition increases the relative impact of sequencing artefacts and background contamination, including signals derived from laboratory reagents. Consequently, rigorous experimental controls and statistical frameworks tailored for low-biomass microbiome data are essential to ensure reliable interpretation [76].

### 7.1. Integrated Multi-Omics Frameworks in Airway Disease

Unlike isolated transcriptomic snapshots, integrative multi-omics captures multi-layered biological information that reflects dynamic host–environment–microbiota interactions. Incorporating extracellular vesicle (EV) omics into this framework is crucial, as EVs serve as specialised vehicles for inter-kingdom protein and RNA exchange within the MuMi layer [77,78]. By combining data from microbial metagenomics/metatranscriptomics with host transcriptomics, proteomics, and metabolomics, researchers can uncover co-regulated molecular modules and putative causal pathways implicated in disease phenotypes and endotypes. For example, in asthma, integrated microbiome and lipidomic profiling revealed associations between airway microbes, sphingolipid/glycerophospholipid metabolism, and type 2 inflammation, highlighting metabolic pathways that may mediate host–microbiota immune responses [79].

A comprehensive review of respiratory microbiome multi-omics highlights the power of combining diverse datasets—from metagenomics and transcriptomics to metabolomics and proteomics—to achieve deeper insights into host–microbe crosstalk and pathogenesis across chronic and acute lung diseases. Such integration helps move beyond associations toward a mechanistic understanding, identifying candidate biomarkers and pathways for targeted intervention [11,80].

### 7.2. Systems Approaches Correlating Molecular Data with Clinical Phenotypes

Embedding multi-omics data from MuMi layer within systems biology frameworks can enable correlation of molecular signatures with key clinical metrics, including FeNO levels, eosinophil counts, lung function indices, and imaging markers. This strategy supports the identification of molecular endotypes—subgroups of patients defined by underlying biology rather than solely by clinical symptoms—which may explain heterogeneity in disease progression and treatment responses. In asthma, for instance, multi-omics analyses have been used to classify patient subtypes based on integrated microbial and metabolic profiles, yielding distinct endotypes with differential immune and metabolic signatures relevant to inflammatory phenotypes [79].

Similarly, in COPD, multi-omics integration of host transcriptome, metabolome, sputum microbiome, and proteome has revealed potential mechanistic links between microbiome metabolic activity and host inflammatory pathways, suggesting that microbial metabolites—such as indole-3-acetic acid—can modulate key immune signalling (e.g., IL-22) and influence epithelial apoptosis and airway inflammation. These multilevel data must be interpreted in light of the lung’s topographical and physiological landscape, as metabolic niches are strictly shaped by regional gradients in oxygen and pH. They also demonstrate how stratified -omic data can illuminate host–microbiota interactions that mediate disease endotypes, including eosinophilic and neutrophilic inflammation [81].

Although the MuMi framework is still conceptually emerging, several classes of molecules may potentially serve as biomarkers associated with this ecosystem. These include microbial community signatures EVs cargo (such as small RNAs and proteins), and host-derived mediators linked to mucosal immunity. Future integrative multi-omics studies will be necessary to validate their clinical relevance [82]. Advanced computational tools—including machine learning and network analyses—are increasingly used to integrate and reduce the dimensionality of multi-omic datasets, enabling the discovery of predictive molecular signatures that correlate with clinical outcomes or treatment responses. By aligning clinical phenotypes with multi-omic endotypes, it becomes feasible to stratify patients, anticipate disease trajectories, and tailor therapeutic strategies accordingly [83].

### 7.3. Towards a “Muco-Microbiotomic Precision Medicine”

The concept of “muco-microbiotomic precision medicine” extends traditional precision medicine by explicitly incorporating the MuMi layer as a central determinant of disease phenotype and therapeutic response. In this perspective, airway diseases are no longer interpreted solely through host-centric inflammatory pathways but rather as outcomes of dynamic interactions within the airway surface ecosystem, in which host cells, microbial communities, and molecular mediators converge. By leveraging EVs as stable ‘liquid biopsies’ in clinically accessible samples, such as induced sputum, it becomes possible to capture real-time signatures of mucosal remodelling and immunological set points. The growing application of integrative multi-omics approaches in respiratory research provides the methodological basis for capturing this complexity and translating it into clinically meaningful stratification models [11].

Building on the evidence discussed in earlier sections—particularly the divergent transcriptomic landscapes observed in eCOPD and eSA—multi-omics integration enables the simultaneous interrogation of host transcriptomics, microbial metatranscriptomics, proteomics, lipidomics, and metabolomics, revealing coordinated molecular programs that underlie apparently similar clinical phenotypes. In asthma, for instance, integrated microbiome–metabolome analyses have identified severe disease endotypes characterised by specific bacterial dysbiosis and lipid metabolic alterations, highlighting how microbial functional activity and host metabolic pathways jointly shape type-2 inflammatory responses [84].

Similarly, in COPD, comprehensive multi-omics studies combining airway microbiome profiling, host transcriptomics, and metabolomics have demonstrated that microbial metabolic products can modulate epithelial apoptosis and immune signalling pathways, reinforcing the concept that chronic airway inflammation emerges from reciprocal host–microbiota interactions rather than from isolated inflammatory axes [81].

A key translational advantage of this framework is its ability to link molecular signatures arising within the MuMi layer to clinically accessible inflammatory and functional readouts, including FeNO, peripheral blood eosinophil counts, lung function parameters, and imaging features. In this regard, a previous study showed that patients with eSA and eCOPD may exhibit comparable peripheral eosinophilia but significantly different FeNO levels, suggesting that similar clinical phenotypes can reflect distinct underlying molecular and regulatory programs [13]. Within a muco-microbiotomic framework, such discrepancies can be interpreted as downstream manifestations of differences in epithelial transcriptional regulation, microbial activity, and vesicle-mediated signalling at the airway surface.

From a therapeutic perspective, muco-microbiotomic precision medicine envisions individualised management strategies guided by integrated -omic signatures rather than single biomarkers. By embedding multi-layer molecular data within systems-biology and computational frameworks, it becomes possible to define biologically grounded endotypes that may predict treatment responsiveness, disease progression, or therapeutic resistance. In asthma, this paradigm is already emerging through multi-omic-based endotyping approaches that combine molecular profiling with clinical phenotypes to guide targeted interventions [73]. Extending this strategy to explicitly include the structure and function of the MuMi layer provides a coherent pathway for precision modulation of inflammation, microbiota-derived signalling, and airway barrier properties, consistent with the complex biology of the respiratory MuMi interface.

**Figure 4:**
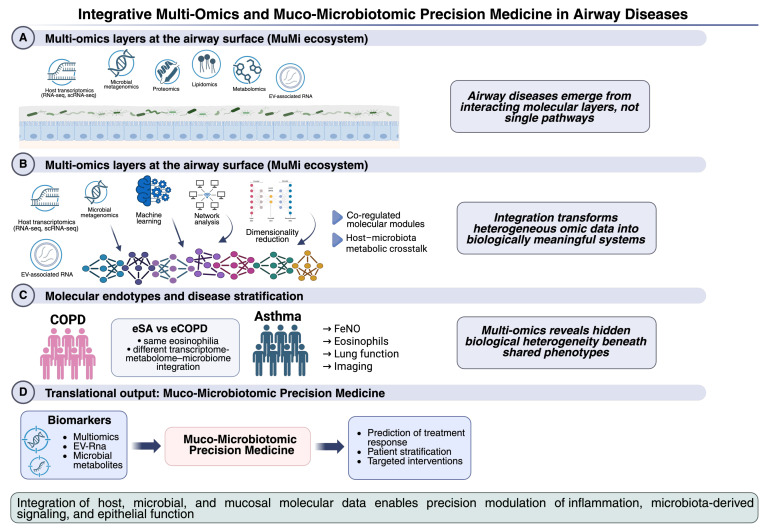
Integrative multi-omics and muco-microbiotomic precision medicine in airway diseases. Schematic overview of how integrative multi-omics approaches connect molecular complexity at the airway surface with clinical phenotypes and translational outcomes. (**A**) The airway MuMi interface is characterised by interacting molecular layers derived from host epithelial cells, immune components, microbial communities, and extracellular vesicles. These layers are captured through complementary -omic platforms, including host transcriptomics, microbial metagenomics and metatranscriptomics, proteomics, lipidomics, and metabolomics. (**B**) Integration of multi-omic datasets using systems biology, network-based analyses, and machine learning enables the identification of co-regulated molecular modules and putative causal pathways that reflect host–microbiota metabolic and inflammatory crosstalk. (**C**) Multi-omics integration reveals molecular endotypes within clinically defined airway diseases such as asthma and COPD, explaining biological heterogeneity underlying shared phenotypes and enabling correlation with clinical metrics including FeNO, eosinophil counts, lung function, and imaging features. (**D**) By embedding muco-microbiotomic data within translational frameworks, integrative multi-omics supports biomarker discovery, patient stratification, and prediction of therapeutic responses, advancing a precision medicine paradigm that accounts for the dynamic interactions shaping the respiratory mucosal ecosystem. Created with https://app.biorender.com/ (accessed on 19 January 2026).

Together, these findings illustrate how integrative multi-omic approaches enable a system-level understanding of airway diseases by linking host, microbial, and mucosal molecular layers to clinically relevant phenotypes. As summarised in Figure 4, the integration of transcriptomic, microbial, proteomic, lipidomic, and metabolomic data supports the identification of biologically grounded disease endotypes and provides a conceptual framework for translating muco-microbiotomic complexity into precision diagnostic and therapeutic strategies.

## 8. Clinical and Therapeutic Implications

Beyond its conceptual relevance, the MuMi layer perspective has concrete implications for both biomarker development and therapeutic innovation in airway diseases. As discussed in earlier sections, alterations in the respiratory mucosal environment—including transcriptomic programs, microbial community structure, and EVs signalling—are closely tied to clinical phenotypes such as eosinophilia and FeNO. These molecular signatures can be harnessed as non-invasive biomarkers that not only reflect disease presence, phenotype, and severity but also provide prognostic and predictive value for disease progression and for responses to both pharmacological and non-pharmacological treatments. Specifically, host-derived EVs from non-invasive samples, such as induced sputum, have emerged as highly stable ‘liquid biopsies’ that reflect the activation state of the airway epithelium and the underlying immunological set point. Moreover, EV-miRNAs circulating in blood or present in airway secretions have been shown to correlate with lung function and inflammatory pathways, supporting their potential utility in early diagnosis and longitudinal monitoring of respiratory diseases [18,85].

From a therapeutic standpoint, integrating knowledge of the MuMi layer opens avenues for novel intervention strategies that extend even beyond conventional pharmacological regimens. One emerging area is microbiota-based therapies that aim to modulate the respiratory or gut microbiome to influence host immunity and inflammatory responses. Although most clinical evidence to date arises from gut–lung axis studies, probiotic and prebiotic interventions have shown promise in modifying systemic inflammation and immunoglobulin profiles in chronic airway conditions, and direct interaction of probiotics with respiratory epithelial cells may enhance the effectiveness of localised microbiome modulation [86]. However, the efficacy of such interventions is likely shaped by the topographical gradients of oxygen and pH that define microbial niches along the respiratory tract. The development of engineered microbial therapies or symbiotic formulations that target specific dysbiotic patterns in the airway microbiota remains a compelling research frontier.

Another promising class of interventions comprises RNA-based therapeutics that directly modulate inflammatory or fibrotic gene networks within the respiratory mucosa. Advances in inhaled RNA delivery, including miRNA mimics, siRNAs, and other oligonucleotide drugs, have shown potential in preclinical models of lung disease, offering high specificity for disease-relevant targets with the theoretical advantage of minimising systemic exposure [87]. These approaches could be further optimised by using engineered EVs as delivery vehicles. EVs naturally bypass complex mucus barriers and target specific niches. As a result, these methods could be tailored to correct maladaptive transcriptomic signatures identified through multi-omics profiling. This approach aligns mechanistic insights with therapeutic design.

In parallel, the field of EVs therapeutics is rapidly expanding. EVs themselves, including exosomes derived from stem cells or engineered to carry specific molecular payloads, are being investigated as targeted delivery vehicles for anti-inflammatory and regenerative cargos in respiratory disease. Inhalable EV formulations are under exploration for conditions ranging from acute lung injury to COPD, with preclinical evidence suggesting favourable immunomodulatory and reparative effects when delivered directly to the airways [88]. Crucially, these vesicles can also transport pro-fibrotic factors, such as TGF-β, making them key targets for interventions aimed at halting structural remodelling, including subepithelial fibrosis and goblet cell hyperplasia. By combining the inherent cell-to-cell communication capabilities of EVs with targeted molecular cargo, these platforms have the potential not only to ameliorate symptoms but also to modify underlying disease pathways.

Emerging antimicrobial strategies, such as engineered bacteriophage therapy, hold significant potential to inform clinical approaches to lung dysbiosis. Engineered phages can be tailored through genetic and chemical modifications to broaden host range, enhance lytic efficacy, and degrade biofilms—features that are especially relevant in chronic airway conditions where impaired mucus clearance and microbial dysbiosis promote persistent infection and inflammation [89]. By targeting specific bacteria without broadly disrupting commensal communities, phage-based interventions could help preserve or restore the integrity of the MuMi layer, thereby reducing pathogen-driven mucosal damage and mitigating the risk of secondary infection. Moreover, advances in delivery technologies, including inhalable formulations designed to navigate airway mucus and reach deep respiratory niches, may improve phage retention and activity at sites of infection while minimising systemic exposure [90].

Together, biomarker development and these emerging therapeutic modalities illustrate a future in which the muco-microbiotomic signature of an individual patient—encompassing mucus rheology, microbial ecology, transcriptomic programs, and vesicular signalling—can guide personalised clinical decisions. This precision medicine paradigm would seek not only to treat symptoms more effectively, but also to intervene upstream in the disease process, targeting the biological interfaces that drive chronic airway inflammation and remodelling.

## 9. Challenges, Future Directions and Conclusions

The recognition of the MuMi layer as a distinct morpho-functional interface at the airway mucosal surface opens new conceptual and translational opportunities but also raises several methodological and interpretative challenges that must be addressed to fully exploit its clinical potential. One of the primary challenges concerns the standardisation of sampling strategies and EV isolation protocols. Respiratory samples such as BAL fluid, induced sputum, nasal lavage, or EBC differ substantially in cellular composition, microbial biomass, and dilution effects, all of which can profoundly influence downstream transcriptomic and vesicular analyses. Furthermore, because the MuMi layer is shaped by topographical gradients of oxygen and pH, the choice of sampling site is critical to ensure that molecular and microbial data accurately reflect regional metabolic niches. In EV research, variability in isolation methods and insufficient control of contaminants remain major obstacles to reproducibility, underscoring the importance of adhering to established guidelines, such as international recommendations for EV characterisation [10].

A second major challenge lies in integrating and causally interpreting multi-omic data derived from the MuMi layer. While advances in high-throughput sequencing and mass spectrometry technologies now allow simultaneous profiling of host transcriptomics, microbial metatranscriptomics, proteomics, lipidomics, and metabolomics, the biological interpretation of these complex datasets remains nontrivial. Distinguishing correlation from causation, particularly in low-biomass environments such as the lower airways, requires robust bioinformatic pipelines, careful experimental design, and validation across independent cohorts. Systems biology and network-based approaches are increasingly employed to address these challenges, yet translating multi-omic associations into mechanistic insights and actionable clinical targets remains an ongoing area of development [11].

An additional challenge concerns the computational and statistical integration of multi-omic datasets derived from low-biomass respiratory samples. In such contexts, microbial and molecular signals are often close to the limits of detection, increasing the risk of background contamination, stochastic noise, and spurious correlations when combining transcriptomic, metatranscriptomic, and EV-derived datasets. Robust analytical pipelines, appropriate contamination controls, and careful normalisation strategies are therefore essential. Moreover, findings generated through integrative multi-omic approaches require validation in large, well-powered and independently replicated cohorts to ensure that identified molecular signatures truly reflect biologically meaningful MuMi-related processes rather than analytical artefacts.

Critically, progress toward clinical implementation will depend on the availability of well-designed clinico-translational studies conducted in patient cohorts with clearly characterised phenotypes. As highlighted in earlier sections, clinically similar phenotypes—such as eSA and eCOPD—may conceal fundamentally distinct molecular programs. The use of EVs as stable ‘liquid biopsies’ may bridge this gap, as their cargo provides a protected snapshot of the mucosal immunological set point that is less prone to the degradation seen in free-floating RNAs.

Bridging molecular signatures within the MuMi layer to clinical outcomes, therefore, requires longitudinal studies that integrate omic data with standardised clinical endpoints, including inflammatory biomarkers (e.g., FeNO, blood eosinophils), lung function, imaging, and treatment response [13].

Despite these challenges, we believe the MuMi layer paradigm represents a significant conceptual advance in respiratory biology. By reframing the airway surface as a dynamic, stratified ecosystem—rather than a passive barrier—this model integrates mucus biophysics, microbial ecology, and vesicle-mediated molecular communication into a unified framework of airway homeostasis and disease. Within this framework, transcriptomics and metatranscriptomics serve as critical bridges between microbial functional activity and host inflammatory and remodelling responses, enabling a functional interpretation of host–microbe interactions that goes beyond taxonomic descriptions alone [11].

Looking forward, the convergence of transcriptomic technologies, EV biology, and multi-omics integration holds promise for redefining disease classification and management in respiratory medicine. By anchoring precision medicine strategies to the biology of the MuMi layer, it may become possible to move from symptom-based or single-biomarker approaches toward biologically grounded endotyping, capable of guiding personalised interventions that target inflammation, microbial signalling, and airway barrier function in a coordinated manner. In this sense, the MuMi layer provides not only a new morpho-functional model of respiratory pathophysiology but also a conceptual scaffold for the next generation of precision pulmonology.

## Figures and Tables

**Table 1:** Metatranscriptomic signatures in chronic respiratory diseases. This table summarises key metatranscriptomic findings reported in major chronic respiratory diseases. For each condition, representative studies are listed together with their principal functional signatures, including microbial transcriptional activity, metabolite-associated pathways, host inflammatory or remodelling responses, and microbe–host interaction networks. Only peer-reviewed PubMed-indexed studies with validated sample processing and RNA-sequencing pipelines were included. Abbreviations: COPD, chronic obstructive pulmonary disease; ISGs, interferon-stimulated genes.

Disease	Key Metatranscriptomic/Functional Findings	Reference (PMID)
COPD	Transcriptionally active *Streptococcus*, *Rothia*, *Pseudomonas* linked to high bacterial biomass, Th17 immune response, exacerbation risk	[40]
COPD (biopsy)	Microbial RNA (very low abundance) associated with host unfolded protein response, microRNA-155 signaling	[45]
COPD (multi-omic)	Microbial metabolites (butyrate, homocysteine, palmitate) interact with host gene signatures, suggesting metabolic crosstalk	[41]
Asthma	Nasal and bronchial microbiome genera (*Moraxella*, *Alloiococcus*) correlate with host inflammatory and ciliary function transcripts; possible protective vs. pathogenic roles	[43]
Viral/Post-infection (e.g., RSV)	Combined metatranscriptome of virus, microbiome and host reveals upregulation of innate immunity, inflammasome pathways, and downregulation of fatty acid metabolism in host	[44]
Early airway injury in smokers	Lower airway dysbiosis (oral commensals) with host transcript signatures (IL-17, IL-6, PI3K, mucin genes)—suggests microbial contribution to inflammation	[42]

## Data Availability

Not applicable.

## Abbreviations

The following abbreviations are used in this manuscript:

ARDS	Acute Respiratory Distress Syndrome
BAL	Broncho-alveolar lavage
COPD	Chronic obstructive pulmonary disease
eCOPD	Eosinophilic Chronic Obstructive Pulmonary Disease
eSA	Eosinophilic Severe Asthma
EBC	Exhaled Breath Condensate
EV	Extracellular Vesicle
FeNO	Fractional exhaled Nitric Oxide
IAV	Influenza A virus
IPF	Idiopathic Pulmonary Fibrosis
IL	Interleukin
KRT-17	Keratin-17
mRNA	messenger Ribonucleic Acid
miRNA	micro Ribonucleic Acid
MuMi	Muco-Microbiotic
OMV	Outer membrane vesicles
PI3K	Phosphoinositide 3-Kinase
RNA	Ribonucleic Acid
sRNA	small Ribonucleic Acid
tRNA	transfer Ribonucleic Acid
